# A metabolomics approach used to profile plasma from portal–arterial pigs revealed differences between breads incurred by dietary fibre and protein contents

**DOI:** 10.1017/jns.2014.14

**Published:** 2014-08-22

**Authors:** Kirstine Lykke Nielsen, Mette Skou Hedemann, Helle Nygaard Lærke, Henry Jørgensen, Knud Erik Bach Knudsen

**Affiliations:** 1 Department of Forensic Medicine, Forensic Chemistry, Aarhus University, Brendstrupgårdsvej 100, DK-8200 Aarhus N, Denmark; 2 Department of Animal Science, Aarhus University, Blichers Allé 20, DK-8830 Tjele, Denmark

**Keywords:** Arabinoxylan, β-Glucan, Catheterised pigs, Rye, Metabolomics, AX, arabinoxylan, BCAA, branched-chain amino acid, BG, β-glucan, ΔAV, portal–arterial difference, DF, dietary fibre, GR, dark ground rye, LC-MS, liquid chromatography–MS, lysoPC, lysophosphatidylcholine, PCA, principal components analysis, RK, rye kernels, WF, white wheat

## Abstract

A liquid chromatography–MS (LC-MS) metabolomics analysis of plasma from portal–arterial catheterised pigs fed breads prepared with whole-grain rye or wheat flour with added concentrated arabinoxylan (AX) or β-glucan (BG) was conducted. Comparison of the effects of concentrated fibres with whole grains has received little attention. Six female catheterised pigs were given two white wheat breads with wheat AX or oat BG, two rye breads with ground rye (GR) or intact rye kernels (RK), and a control white wheat bread (WF) on separate occasions in a randomised cross-over design. The amount of available carbohydrate was similar for the five breads but varied in the content of protein. Plasma was collected continuously for 4 h after feeding. Glucose levels in the portal vein were reduced postprandially in response to the AX, GR and RK breads that had high contents of AX compared with WF bread (*P* < 0·03). AX and RK breads further tended to decrease plasma levels of some lysophosphatidylcholine species (*P* ≤ 0·10). The abundance of amino acids in plasma correlated with the protein contents in the breads and leucine uptake significantly affected insulin secretion in the mesenteric artery. In conclusion, the present study revealed that concentrated AX in wheat bread had similar positive effects as whole-grain rye bread on glucose and lipid metabolism.

Whole-grain consumption is associated with a reduced risk of a number of diseases related to Western lifestyle such as obesity^(^^1^^,^^2^^)^, CVD^(^^3^^)^ and type 2 diabetes^(^^4^^)^. Whole grains contain cereal dietary fibres (DF) like arabinoxylan (AX) and (1 → 3)(1 → 4)-β-d-glucan (referred to as β-glucan; BG) that are known to attenuate postprandial blood glucose and insulin levels and lower serum cholesterol^(^^5^^–^^8^^)^; these are functionalities that have potential health beneficial effects.

Our knowledge of how DF regulates events at the molecular level is still limited and understanding the biochemical mechanisms behind the protective effects may lead to development of new health-promoting products with specifically designed fibre fractions. Combining nutriomic techniques like metabolomics with traditional measurements of biomarkers such as glucose and insulin levels may provide such information. High-DF/rye diets have shown to alter the concentrations of betaine and its related metabolites in the homocysteine metabolism in plasma of human subjects^(^^9^^,^^10^^)^ and animal models^(^^11^^–^^14^^)^, which may be associated with a reduced risk of CVD. Differences in plasma metabolites of branched-chain amino acids (BCAA), aromatic amino acids and lysophosphatidylcholine (lysoPC) species, together with some precursors and breakdown products, have furthermore been found in response to high-DF/rye diets that may mediate positive effects related to reduced risks of obesity and diabetes^(^^10^^,^^15^^,^^16^^)^. In the above and other reported studies, the subjects had been adapted to the diets for at least 7 d.

In the present study the porcine portal–arterial catheterisation model was used to study the acute metabolic response, similar to what is done when measuring glycaemic index in human subjects without any prior adaptation, to five test breads with different fibres and fibre levels. The test breads were a regular white wheat (WF) bread, two WF breads supplemented with either concentrated wheat AX or concentrated oat BG, a dark ground rye (GR) bread, and a rye bread with intact rye kernels (RK). Concentrated AX and BG have often proved their effects in comparison with low-fibre diets. It is, however, important to evaluate the clinical importance of concentrated DF relative to whole-grain DF, which has received little attention. As such, this design allowed for comparison of whole-grain/whole-kernel *v.* concentrated DF as well as a comparison of high fibre *v.* low fibre. The aim was to determine the properties of concentrated AX, BG, whole-grain rye and intact RK on glycaemic control using an untargeted liquid chromatography–MS (LC-MS) metabolomics approach.

## Experimental methods

### Animal model

A group of six female pigs (crossbreeds of Duroc × Danish Landrace × Yorkshire from the swineherd at Aarhus University, Foulum, Tjele, Denmark) with an initial body weight of 60·2 (sem 3·1) kg were catheterised in the mesenteric artery and the portal vein using the method described by Jørgensen *et al.*^(^^17^^)^. Pigs were selected for surgery if they had a plasma glucose level below 5·8 mmol/l at 1 h after ingesting a traditional swine diet, and if they would eat rye bread during testing. The portal vein catheter was infused with 0·5 litres sterile saline on the first postoperative day to keep the fluid balance stable after surgery and keep the catheter patent and the pigs were exercised for 3 d postoperative. In total, 7–9 d were spent to recover. Catheters were flushed aseptically with 1000 IU/ml of heparin solution every 3–4 d and otherwise if needed to maintain patency. Catheters were secured using pouches attached to the side of the pig using Leucoplast/Tensoplast (BSN Medical) and an elastic tubular net. The pigs were kept individually in pens with a concrete floor. Elevated plastic grids covering half of the pen allowed the pigs to rest and stay dry.

The animal experiment was conducted according to the license obtained by the Danish Animal Experiments Inspectorate, Ministry of Food, Agriculture and Fisheries, Danish Veterinary and Food Administration.

### Breads

The three commercial breads were produced at Lantmännen Schulstad A/S (Hammerholmen). Commercial names are: Sundbrød Hvede Toast (WF), Mørkt Rugbrød (GR) and Levebrød Multikernerugbrød (RK). The AX and BG breads were baked in-house. AX bread was made from white wheat flour (678 g/kg), wheat AX concentrate (244 g/kg; Manildra Group), water, baker's yeast (35 g/kg), sugar (9 g/kg), salt (17 g/kg) and shortening (18 g/kg). Soluble wheat AX was isolated from the soluble fraction after extraction of starch and gluten, concentrated by evaporation, heat treated, further treated with α-amylase and glucoamylase, precipitated with ethanol (1:3, v/v), filtered and finally dried on a spray dryer. BG was made from white wheat flour (710 g/kg), Promoat™ (133 g/kg; Biovelop AB), Vitacel^®^ WF 600 (69 g/kg; Rettenmaier and Söhne GmbH), wheat gluten (10 g/kg), baker's yeast (35 g/kg), sugar (9 g/kg), salt (17 g/kg) and shortening (18 g/kg). Soluble BG was obtained from the sub-aleurone of oat by combining wet-milling and enzymic hydrolysis. Since a high dose of BG from Promoat™ would cause the dough to be very sticky, the amount was reduced and cellulose/purified wheat fibre (Vitacel^®^) was added to balance the DF content in the BG bread relative to the other high-DF breads. Details concerning molecular weight and molecular characteristics of the DF concentrates can be found in the papers of Kasprzak *et al.*^(^^18^^,^^19^^)^.

### Design

Pigs were fed each of the five breads in a randomised 5 × 6 incomplete Latin square cross-over design. On sampling days the morning meal was replaced with experimental bread (Mondays and Thursdays, 3–4 d apart) that provided about 200 g of available carbohydrate and blood was collected in heparinised Vacutainers (Greiner Bio-One) from the mesenteric artery and portal vein. Plasma for metabolomics analysis was collected at –15 (fasting value, t_0_), 30, 60, 90, 120, 180 and 240 min postprandial. After collection, catheters were flushed with 5 ml 0·9 % sterile saline to replace fluid loss and filled with 100 IU/ml heparin solution to prevent clotting. Packed cell volume values were measured at –15 and 240 min. Blood was centrifuged at 2000 ***g*** for 12 min at 4°C and plasma was kept frozen at –80°C until further analysis. Between interventions the pigs were fed a washout diet low in DF consisting of wheat flour (807 g/kg), Lacprodan^®^-87 (73 g/kg; Arla Foods Ingredients), rapeseed oil (30 g/kg), Vitacel^®^ WF 600 (Rettenmaier and Söhne GmbH) and a vitamin/mineral mixture containing synthetic amino acids providing all the necessary vitamins and minerals. Pigs were fed three times per d with 635 g of washout diet per meal. When reaching 70 kg of body weight the dose was 750 g. Once per week, the pigs were weighed and received an intramuscular supplement of 400 mg Fe^3+^ (Uniferon^®^; Pharmacosmos A/S). The pigs had free access to water during the entire study period.

### Analytical methods

Test breads and the washout diet were freeze-dried and ground to a particle size less than 0·5 mm for chemical analyses. All analyses were performed in duplicate. DM content was determined by oven drying at 103°C for 20 h. Gross energy was analysed by use of an oxygen bomb calorimeter (Parr Instrument Company). Ash was analysed by an AOAC method^(^^20^^)^. Protein (N × 6·25) was measured by Dumas^(^^21^^)^. Amino acids were quantified after hydrolysis for 23 h at 110°C with or without performic acid oxidation using ion exchange chromatography and photometric detection after ninhydrin reaction^(^^22^^)^. Fat was extracted with diethyl ether after HCl hydrolysis according to the Stoldt procedure^(^^23^^)^. Starch and NSP were analysed essentially as described by Bach Knudsen^(^^24^^)^; 2 m-H_2_SO_4_ for 1 h was used instead of 1 m-H_2_SO_4_ for 2 h for the NSP analysis. The content of non-digestible carbohydrates (NDC) was determined by direct acid hydrolysis without starch removal and alcohol precipitation. The total NDC was calculated by subtraction of the starch content. AX was calculated as the sum of arabinose and xylose from the NSP analysis. BG was analysed by the enzymic–colorimetric method of McCleary & Glennie-Holmes^(^^25^^)^. Klason lignin was measured gravimetrically as the sulfuric acid-insoluble residue as described by Theander & Åman^(^^26^^)^. The content of available/digestible carbohydrates was calculated as:
(1)
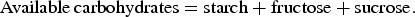


Chemical compositions of the test breads are presented in Table 1.

**Fig. 1.**
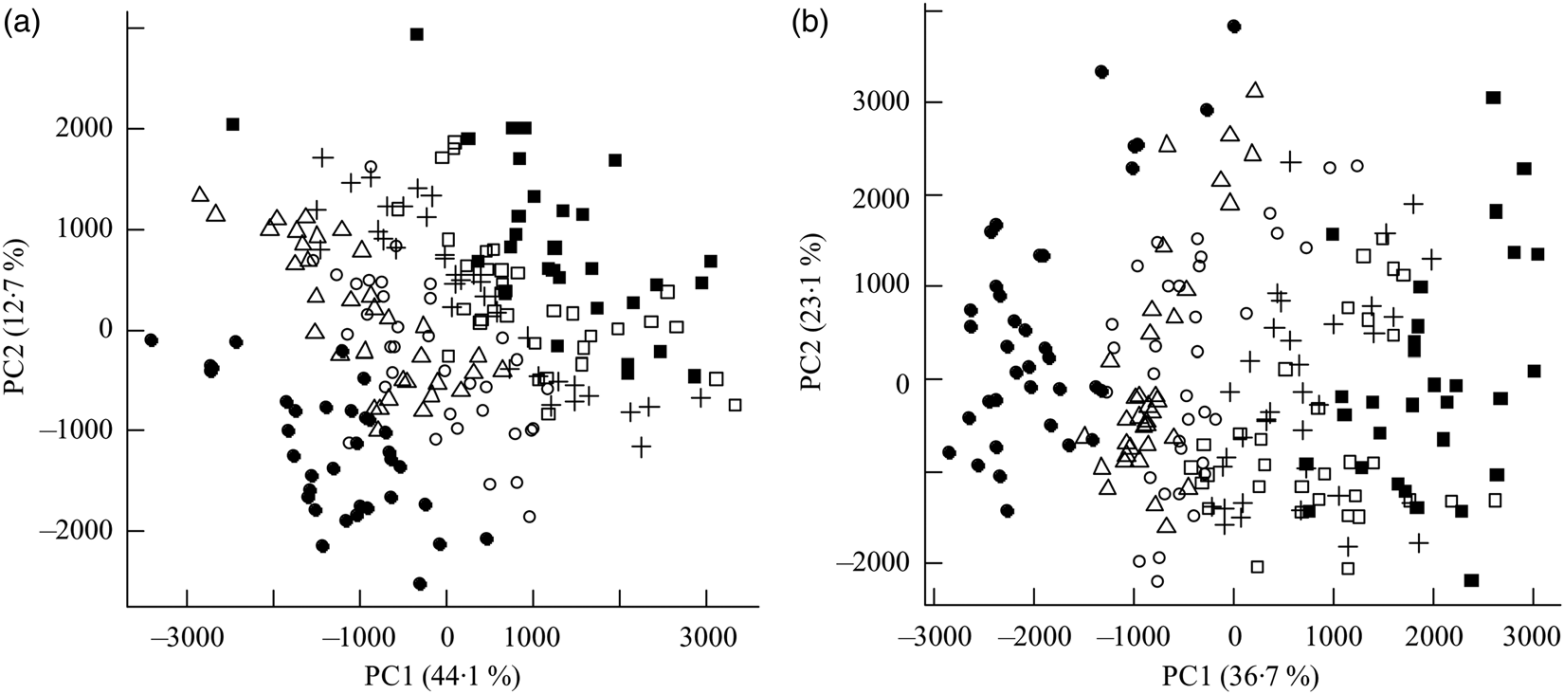
Principal components analysis scores plot of plasma from the mesenteric artery (a) and the portal vein (b) of pigs taken at 0, 30, 60, 90, 120, 180 and 240 min after consumption of five different test breads. ■, Fasting values; Δ, white wheat bread; ●, arabinoxylan bread; ○, β-glucan bread; +, dark ground rye bread; □, rye bread with kernels. The amount of total variation accounted for by the principal components PC1 and PC2 is shown in parentheses.

**Table 1.** Chemical composition, amount of available carbohydrate (CHO) per meal and insulinaemic index (II, 120 min, using the white wheat (WF) bread as the reference) of the experimental test breads

	Bread				
Chemical composition (g/kg DM)	WF	AX	BG	GR	RK
DM (g/kg as-fed basis)	634	708	615	520	543
Gross energy (MJ/kg DM)	18	19	18	18	18
Ash	24	42	29	36	32
Protein (N × 6·25)	132	210	122	95	91
Leucine	8	12	7	5	5
Valine	5	9	5	4	4
Tyrosine	3	6	3	2	2
Tryptophan	1	2	1	1	1
Phenylalanine	6	8	5	4	4
Fat	34	28	31	22	23
Starch	711	514	612	588	608
BG	3	3	52	21	19
AX	17	78	32	76	77
Dietary fibre (NDC + lignin)	77	212	199	209	220
Available CHO (g/meal)	210	225	229	210	211
II (%)					
Mean	100	93	79	61	78
sd		17	17	17	17

AX, arabinoxylan; BG, β-glucan; GR, dark ground rye; RK, rye kernels; NDC, non-digestible carbohydrates.

Plasma insulin was analysed by time-resolved fluoroimmunoassay as described by Løvendahl & Purup^(^^27^^)^ and the insulinaemic indices were calculated from the 120 min incremental area under the insulin curves using the WF bread as the reference.

### Reversed-phase HPLC-MS

Plasma samples (200 µl) were mixed with 20 µl 100 µg/ml internal standard mix of glycocholic acid (glycine-1-^13^C) and lysoPC (17:0). The samples were added to 600 µl ice-cold methanol, immediately vortexed and incubated at 4°C for 20 min for deproteinisation. The supernatant fractions were collected after centrifugation at 13 200 ***g*** for 10 min and evaporated to dryness under a stream of N_2_. The resulting dry residues were re-suspended in 200 µl water–acetonitrile–formic acid (95:5:0·1, by vol.) and centrifuged at 10 621 ***g*** for 10 min. The supernatant fractions were injected into the LC-MS.

For the LC-MS analysis, an Ultimate 3000 (Dionex) HPLC system was coupled to a MicrOTOF-Q II mass spectrometer (Bruker Daltonik GmbH) operating in positive electrospray ionisation mode. The scan range was from 50 to 1000 *m/z* at a sampling rate of 2 Hz. The capillary voltage was 4500 V, the nebulising gas pressure was 1·8 bar and the drying gas flow and temperature were 8·0 litres/min and 200°C, respectively. Lithium formate at a concentration of 5 mm in water–isopropanol–formic acid (50:50:0·2, by vol.) was employed as an external calibrant. For MS/MS analysis, Ar was used as the collision gas, and collisions were carried out at energies from 6 to 60 eV. All other parameters were the same as above.

Chromatographic separation was performed on a Discovery^®^ HS C_18_ column (15 cm × 2·1 mm, 3 µm) used together with a C_18_ pre-column. The column was held at 30°C. The injection volume was 5 µl. Mobile phase A consisted of water–formic acid (100:0·1, v/v) and mobile phase B consisted of acetonitrile–formic acid (100:0·1, v/v). The column was equilibrated for 5 min before the gradient started at 10 % B for 1 min and then linearly increased to 100 % B within 20 min. The mobile phase was kept at isocratic conditions (100 % B) for 5 min and then turned back to 10 % B. Total analysis time was 31 min and the flow rate was 200 µl/min. A sample of pooled plasma and blanks was re-injected after each seven samples for quality control.

### Data analysis

Acquired mass spectra were calibrated and converted into mzXML file format using CompassXport (Bruker Daltonik GmbH) to carry out data analysis. MZmine 2·3^(^^28^^)^ was employed for pre-processing of data using a centroid peak detector algorithm and the RANSAC aligner. A matrix was generated with retention time, *m/z* and respective intensities of ions. Data were explored as arterial and portal vein samples, and as portal–arterial differences (ΔAV). Arterial and portal vein data remained non-normalised, whereas the ΔAV were calculated from normalised data, so that arterial intensities of each metabolite could be subtracted from the venous intensities. Normalisation was performed using the standard compound normaliser in MZmine 2·3 with a weighted contribution of all internal standards. Orthogonal signal correction according to Wold *et al*.^(^^29^^)^ was applied using the PLS toolbox for MATLAB 7·11·0 (R2010b) (The MathWorks, Inc.) to selectively remove irrelevant variation between pigs. Data were pareto scaled and principal components analysis (PCA) was performed using LatentiX 2·00 (Latent5 Aps). Outliers were removed based on 95 % CI and plots of residual variance *v.* Hotelling's T^2^ after which the models were recalculated. Loading plots were used to detect metabolite ions with the greatest influence on clustering. Compounds were identified based on database searches in METLIN (http://metlin.scrips.edu), The Human Metabolome Database (http://www.hmdb.ca) and PubChem compound database (http://pubchem.ncbi.nlm.nih.gov) using accurate mass and mass spectrometric fragmentation patterns. Authentic standards were used for confirmation of the metabolites when available.

Effects of breads, time and their interactions as well as sampling site (artery or vein) were analysed as repeated measurements using the MIXED procedure of SAS (SAS, version 9.3; SAS Institute Inc.). Compounds were analysed as a linear mixed model:
(2)
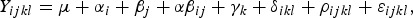

where *Y*_*ijkl*_ is the dependent variable, μ is the overall mean, α_*i*_ is the effect of bread (*i* = WF, GR, RK, BG, AX), β_*j*_ is the time after feeding (*j* = −15 (0), 15, 30, 45, 60, 90, 120, 180 and 240), and α β_*ij*_ is the interaction term. The three terms *y*_k_ (*k* = pig 1,...,), δ_*ikl*_ (*l* = period 1,...,5) and ρ_*ijkl*_ accounted for repeated measurements being performed on the same pig (*y*_*k*_) among periods in the cross-over design (δ_*ikl*_), and on the same pig within a sampling day (ρ_*ijkl*_). ε_*ijkl*_ is the residual error component. The covariance structure of ρ_*ijkl*_ was modelled using the spatial power option, which takes into account the different intervals between repeated measurements. Pearson correlation coefficients were calculated using the CORR procedure of SAS. The correlation of glucose and leucine to insulin was further analysed by a multiple regression correlation analysis using the REG procedure of SAS. If variance homogeneity was not present the data were ln(*x*) transformed before statistical analysis. Statistical significance was set as *P* < 0·05 and 0·05 ≤ *P* <0·10 as trends.

## Results

Orthogonal signal correction-filtered PCA models of arterial samples individually and portal vein samples individually showed similar separation patterns according to intake of the different bread types (Fig. 1). The separations were caused by glucose and amino acids. Significantly higher glucose concentrations were found for the WF bread compared with the AX, GR and RK breads in the portal vein but not in the artery (Table 2). Plasma levels of tyrosine, leucine, phenylalanine and tryptophan were increased in response to the AX bread and decreased in response to the rye breads compared with WF bread. This clearly mirrored the protein and amino acid compositions in the breads, with significant correlations between breads and plasma (Table 2). A correlation between the insulin response and glucose and leucine was also found (Table 2). A correlation between insulin and the other amino acids was not detected. Leucine, however, might influence insulin due to its correlation with glucose uptake (*P* < 0·001), which is why a multiple regression correlation analysis was performed. The isolated effects of leucine were found to affect insulin in the mesenteric artery (*P* = 0·001) but not in the portal vein (*P* = 0·41), whereas the effects of glucose on insulin was significant in both the artery and vein (*P* < 0·001).

**Table 2.** Plasma levels of identified metabolites (30–240 min postprandial) from the mesenteric artery (*A*) and the portal vein (*V*) reflecting differences in pigs (*n* 6) fed arabinoxylan (AX) bread, β-glucan (BG) bread, dark ground rye (GR) bread and rye bread with kernels (RK) relative to pigs fed white wheat (WF) bread (Mean values with their standard errors, and correlations)

				Bread						*P*			Correlation between compound in bread and plasma		Correlation between insulin and compound in plasma	
Compound	Retention time (min)	*m/z*	Mass accuracy (ppm)	WF	AX	BG	GR	RK	sem	Bread	Time	Bread × time	*r*	*P*	*r*	*P*
Glucose*																
[M + Na]^+^	2·29	203·052	5·7													
*A*				1·00	0·96	0·95	0·96	1·01	0·02	0·30	<0·001	0·03	0·20	0·29	0·53	<0·001
*V*				1·00^a^	0·93^b^	0·95^a,b^	0·91^b^	0·93^b^	0·02	0·03	<0·001	<0·001	0·52	0·003	0·44	<0·001
Tyrosine*																
[M + H]^+^	2·77	182·081	0													
[M-H_2_O + 2H]^+^	2·77	165·053	6·1													
[M-COOH]^+^	2·75	136·072	29·4													
*A*				1·00^b^	1·34^a^	0·97^b^	0·71^c^	0·53^c^	0·08	<0·001	<0·001	0·005	0·77	<0·001	0·06	0·37
*V*				1·00^b^	1·51^a^	1·05^b^	0·77^c^	0·73^c^	0·11	<0·001	<0·001	<0·001	0·74	<0·001	0·10	0·16
Leucine*																
[M + H]^+^	3·08	132·097	11·4													
[M-COOH]^+^	3·08	86·097	1·2													
[M + Na]^+^	3·08	154·081	22·1													
*A*				1·00^a,b^	1·10^a^	0·89^b,c^	0·76^d^	0·78^c,d^	0·04	<0·001	<0·001	<0·001	0·77	<0·001	0·19	0·006
*V*				1·00^b^	1·15^a^	0·93^b^	0·80^c^	0·78^c^	0·03	<0·001	<0·001	<0·001	0·86	<0·001	0·22	0·002
Phenylalanine*																
[M + H]^+^	4·01	166·085	7·6													
[M-COOH]^+^	4·01	120·080	11·7													
[M-CH_4_NO_2_]^+^	4·01	103·053	17·5													
*A*				1·00^b^	1·19^a^	1·02^b^	0·81^c^	0·83^c^	0·03	<0·001	<0·001	<0·001	0·43	0·02	0·08	0·24
*V*				1·00^b^	1·21^a^	1·02^b^	0·81^c^	0·75^d^	0·02	<0·001	<0·001	<0·001	0·43	0·02	0·11	0·10
Tryptophan*																
[M + H]^+^	5·60	205·095	13·2													
[M-H_2_N]^+^	5·60	188·070	2·1													
[M-COOH]^+^	5·60	159·090	14·0													
[M-C_2_H_6_NO + 2H]^+^	5·59	146·059	0													
*A*				1·00^b^	1·16^a^	0·98^b^	0·91^b^	0·89^b^	0·05	0·002	<0·001	0·003	0·62	<0·001	0·06	0·42
*V*				1·00^b^	1·23^a^	1·05^b^	0·91^c^	0·83^c^	0·03	<0·001	<0·001	<0·001	0·73	<0·001	0·11	0·13

ppm, Parts per million.

^a,b,c,d^Mean values within a row with unlike superscript letters were significantly different (*P* < 0·05).

*Identification confirmed by standard comparison.

**Fig. 2.**
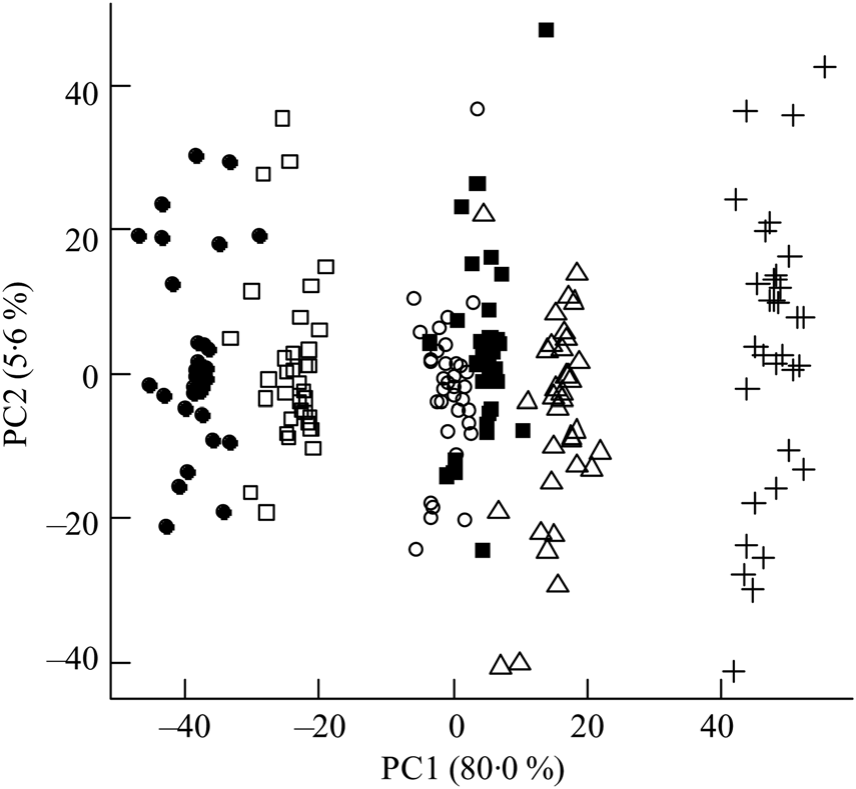
Principal components analysis scores plot of portal–arterial differences in pigs fed five different test breads. Plasma was sampled at 0, 30, 60, 90, 120, 180 and 240 min postprandial. ■, Fasting values; Δ, white wheat bread; ●, arabinoxylan bread; ○, β-glucan bread; +, dark ground rye bread; □, rye bread with kernels. The amount of total variation accounted for by the principal components PC1 and PC2 is shown in parentheses.

The ΔAV were calculated and reflected the net absorption of metabolites from the gastrointestinal tract. The orthogonal signal correction-calibrated PCA model revealed a clear distinction and variation in metabolite absorption due to bread type on principal component 1 (Fig. 2). The two rye breads, with almost similar chemical compositions apart from the milled/intact kernels, were grouped very distantly and RK bread showed a greater resemblance to the AX bread than the GR bread.

**Fig. 3.**
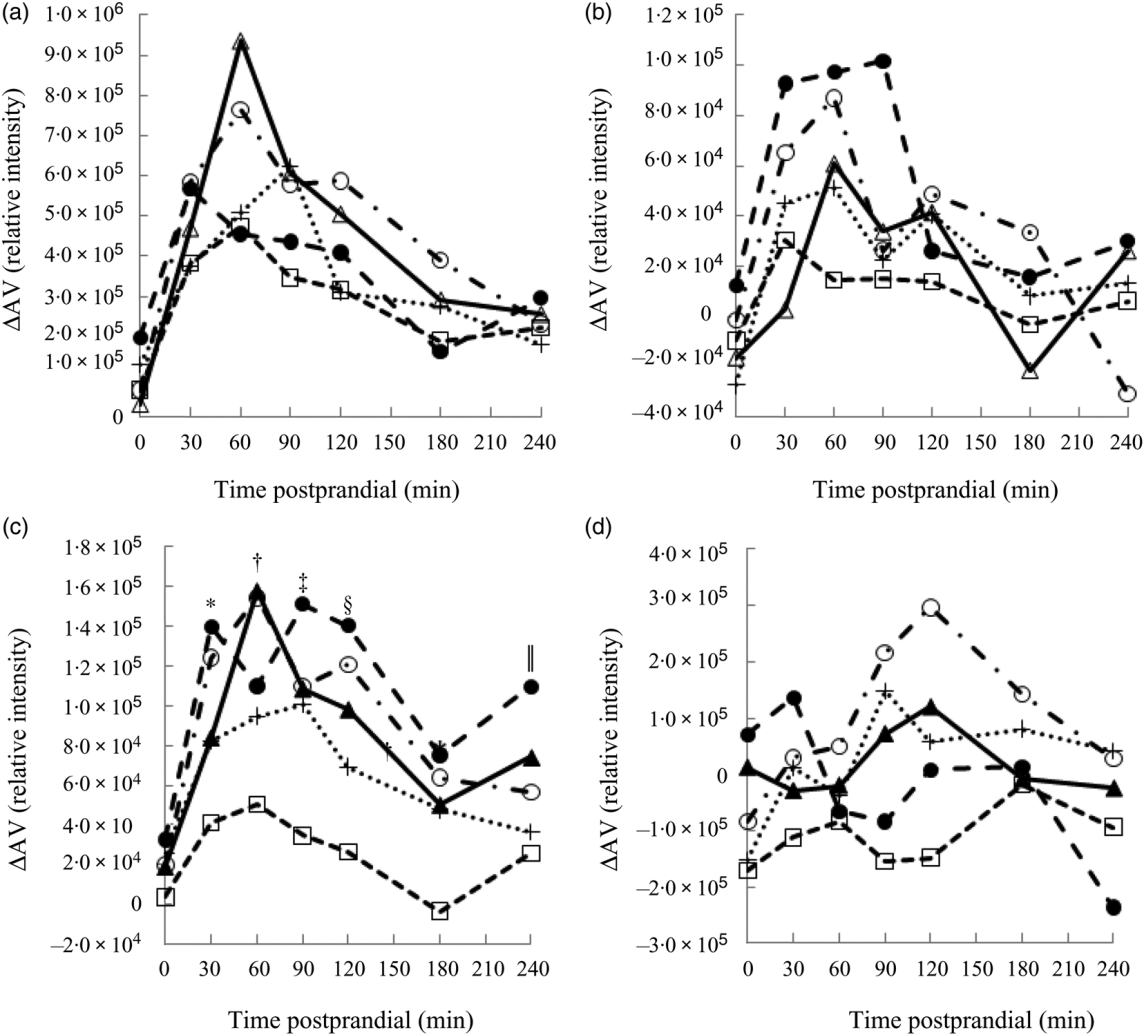
Time profiles of identified plasma metabolites in pigs fed five different test breads: (a) glucose (pooled sem = 8·0 × 10**^4^**); (b) leucine (pooled sem = 1·7 × 10^4^); (c) phenylalanine (pooled sem = 4·1 × 10^4^); (d) lysophosphatidylcholine 18 : 2 (pooled sem = 1·1 × 10^5^). ▴, White wheat bread (WF); ●, arabinoxylan bread (AX); ○, β-glucan bread (BG); +, dark ground rye bread (GR); □, rye bread with kernels (RK). Means at the same time point differ: * RK < AX, BG (*P* < 0·05); † RK < BG, WF (*P* < 0·003); ‡ RK < AX, BG, GR, WF (*P* < 0·05); § GR < AX and RK < AX, BG, WF (*P* < 0·03); ‖ GR, RK < AX (*P* < 0·03). ΔAV, portal–arterial difference.

Metabolites with greatest influence on the clustering were located (Table 3). However, large variations in the ΔAV were found due to the semi-quantitative nature of the data, which caused very few significant bread differences and no clear correlations between plasma and bread contents or insulin. Time profiles of metabolite compounds of ΔAV of glucose, leucine, phenylalanine and lysoPC 18:2 are depicted in Fig. 3. The WF bread had a numerically higher glucose peak than the AX, GR and RK breads, although not significant (Fig. 3(a)) and leucine absorption was numerically higher for AX and BG breads than the other breads (Fig. 3(b)). Absorption of phenylalanine (Fig. 3(c)) and tryptophan (data not shown) was significantly lower for the RK bread compared with the WF-based breads (WF, AX and BG breads). WF, BG and GR breads tended to induce increased levels of lysoPC 18:2, postprandial, whereas AX and RK breads maintained baseline levels or slight decreases (Fig. 3(d)) – similarly for lysoPC 20:4 and 16:0 (data not shown).

**Table 3.** Plasma levels of identified metabolites (30–240 min postprandial) reflecting distinctions in portal–arterial differences in pigs (*n* 6) fed arabinoxylan (AX) bread, β-glucan (BG) bread, dark ground rye (GR) bread and rye bread with kernels (RK) relative to pigs fed white wheat (WF) bread (Mean values with their standard errors, and correlations)

				Bread						*P*			Correlation between compound in bread and plasma		Correlation between insulin and compound in plasma	
Compound	Retention time (min)	*m/z*	Mass accuracy (ppm)	WF	AX	BG	GR	RK	sem	Bread	Time	Bread × time	*r*	*P*	*r*	*P*
Glucose*																
[M + Na]^+^	2·29	203·052	5·7	1·00^a^	0·82^a,b^	1·03^a^	0·78^a,b^	0·65^b^	0·14	0·09	<0·001	0·15	0·25	0·18	0·18	0·008
Glucose dimer*																
[M + Na]^+^	2·29	383·115	4·0													
Valine*																
[M + H]^+^	2·31	118·086	2·5	1·00	0·84	0·87	1·06	0·71	0·19	0·65	0·40	0·35	0·05	0·78	–0·03	0·69
Leucine*																
[M + H]^+^	3·08	132·097	11·4	1·00	2·40	1·80	1·21	0·53	0·79	0·24	<0·001	0·08	0·28	0·13	0·005	0·93
[M-COOH]^+^	3·08	86·097	1·2													
Phenylalanine*																
[M + H]^+^	4·01	166·085	7·6	1·00^a,b^	1·29^a^	1·10^a,b^	0·78^b^	0·30^c^	0·22	0·001	<0·001	0·59	0·21	0·26	0·08	0·23
[M-COOH]^+^	4·01	120·080	11·7													
Tryptophan*																
[M-H_2_N]^+^	5·66	188·070	2·1	1·00^a^	1·20^a^	1·04^a^	0·82^a,b^	0·01^b^	0·45	0·06	0·01	0·57	0·18	0·35	–0·12	0·29
[M-C_2_H_6_NO + 2H]^+^	5·59	146·059	0													
UI PC†	15·74	524·368		1·00	5·93	4·94	0·19	8·46	2·92	0·18	0·06	0·26	–	–	–	–
LysoPC 18 : 0†																
[M + H]^+^	17·60	524·367	8·8	1·00	0·92	0·20	1·18	1·40	0·70	0·73	0·19	0·19	–	–	–	–
LysoPC 20 : 4†																
[M + H]^+^	17·65	544·338	4·2	1·00^a^	0·70^a,b^	1·39^a^	0·94^a^	0·09^b^	0·30	0·06	0·48	0·32	–	–	–	–
LysoPC 18 : 2†																
[M + H]^+^	17·72	520·338	4·4	1·00^a^	0·71^a,b^	1·60^a^	1·03^a^	0·06^b^	0·36	0·06	0·08	0·28	–	–	–	–
LysoPC 16 : 0*†																
[M + H]^+^	19·11	496·338	3·6	1·00^a,b^	0·53^b^	1·82^a^	0·85^a,b^	0·01^b^	0·70	0·10	0·08	0·94	–	–	–	–
LysoPC 18 : 1*†																
[M + H]^+^	19·70	522·354	3·8	1·00	1·31	3·15	1·30	0·05	1·51	0·46	0·04	0·72	–	–	–	

ppm, Parts per million; UI, unidentified; PC, phosphatidylcholine; lysoPC, lysophosphatidylcholine.

^a,b,c^Mean values within a row with unlike superscript letters were different (*P* < 0·10).

*Identification confirmed by standard comparison.

†PC and LysoPC were identified from characteristic MS fragments of 184, 104, and 86 in positive electrospray ionisation mode.

## Discussion

Studies in human subjects only allow collection of peripheral blood in which many metabolites have been metabolised by the liver and other tissues. Consequently, several effects cannot be measured. The porcine portal–arterial catheterisation model allows simultaneous blood draws from the mesenteric artery and the portal vein; portal blood permits measurements of absorbed nutrients and gut hormones from the intestine to the liver, whereas arterial blood represents the systemic circulation. The ΔAV thus represents the enrichment of compounds coming from the gastrointestinal tract^(^^30^^)^. In the present study the acute metabolic effects to five breads were studied in plasma from the porcine portal–arterial catheterisation model. LC-MS metabolomics was applied to gain additional insight into the effects of AX and BG, whole-grain rye and refined white wheat.

The four high-fibre breads had 61–65 % more DF than the WF bread; for AX, GR and RK breads predominantly in form of AX and for BG bread in form of BG. The different breads also incurred significant differences in the physico-chemical properties of digesta^(^^19^^)^. This had an impact on glucose absorption, showing glucose to be attenuated in response to the AX, GR and RK breads. Decreased glucose absorption could be due to delayed gastric emptying and an increased viscosity of digesta caused by the high AX contents as seen in an accompanying study with ileal-cannulated pigs fed the same bread diets by Kasprzak *et al.*^(^^19^^)^. Increased viscosity decreases availability of digestive enzymes to the food bolus, thereby reducing glucose absorption. A reduced glucose uptake causes an attenuated insulin response that may prevent the development of diseases related to the metabolic syndrome. The BG bread was not found to reduce glucose absorption due to degradation of the BG content in the small intestine of the pigs, which was found in the study with ileal-cannulated pigs^(^^19^^)^. Glycaemic index measurements from human subjects with the metabolic syndrome^(^^31^^)^ and quantitative glucose measurements from catheterised pigs^(^^32^^)^ have shown similar reductions in glucose absorption but only significant for the AX bread. The LC-MS data were only semi-quantitative, but the data seemed to mimic the quantitative results, which may validate the metabolomics method.

Plasma levels in the portal vein and the mesenteric artery correlated with the amino acid contents in the breads, showing uptake and concentration to be linked. The AX bread had a high protein content originating from the refined wheat AX concentrate, which explains why AX bread yielded the highest content of amino acids in plasma. Protein levels between the breads, however, also significantly affected the insulin secretion through leucine. The ability of the liver to metabolise BCAA is limited^(^^33^^)^, which could explain why the effects of leucine were evident in the artery but not the portal vein. BCAA, especially leucine, are known to influence insulin responses^(^^34^^,^^35^^)^. Increased plasma levels of BCAA and phenylalanine have furthermore been associated with an increased risk of diabetes^(^^36^^)^. Two other studies with human subjects found leucine and isoleucine to be decreased after 8 or 12 weeks intervention with rye compared with oat and wheat^(^^10^^,^^16^^)^. However, consumption of other food products and differences in protein intake could affect the results in these studies. On the other hand, rye may have a positive effect on BCAA metabolism to lower plasma levels that could be linked to decreased insulin secretion^(^^10^^)^; several studies report rye to lower postprandial insulin responses without affecting glycaemia^(^^37^^–^^39^^)^. Either way, these results warrant a more careful consideration concerning protein contents when investigating carbohydrate metabolism.

The lysoPC species were very dominant from the PCA models of ΔAV; the bread effects found were, however, minor. LysoPC are major plasma lipid components that serve as signalling molecules and as transporters of fatty acids, phosphatidylglycerol and choline^(^^40^^)^, but different lysoPC species may have different functions and may exert different effects. A study with human subjects found increased concentrations of lysoPC during an oral glucose tolerance test^(^^41^^)^. Another study found higher plasma contents of lysoPC after an intervention with wheat, oat and potato compared with rye^(^^16^^)^. This indicates that glucose uptake changes the lipid profile of lysoPC, whereas DF or other bioactive/antioxidant compounds in rye might counteract the effect. Studies investigating acquired obesity have additionally found lysoPC levels in plasma to be increased^(^^42^^,^^43^^)^ and they are major plasma lipid components of oxidised LDL with implications in atherosclerosis and chronic inflammation^(^^40^^,^^44^^)^. Therefore, decreased concentrations may be of interest to reduce the risk of lifestyle diseases. In this case WF, BG and GR breads tended to cause higher concentrations of lysoPC 18:2, 20:4 and 16:0 in plasma of the pigs than AX bread and especially RK bread. The explanation might be due to an attenuated glucose uptake with AX and RK breads. However, this was also true for GR bread. The reason for this difference between GR *v.* RK and AX breads is unknown.

In summary, analysis of the plasma metabolome of portal–arterial catheterised pigs revealed differences in the acute response related to DF and protein contents of the breads. Detection of more DF-related markers was lacking and needs to be optimised in future studies. However, due to the short time span of plasma collection for 4 h postprandial, microbial degradation products, nutritive and non-nutritive (phytochemicals), could not be found in the present study. On the other hand, AX, GR and RK breads were found to efficiently decrease glucose absorption compared with WF bread, most probably due to the high AX contents. This indicates that concentrated AX incorporated into WF bread has similar effects on the glycaemic response as AX present in whole-grain rye bread, and may be useful in the prevention and treatment of obesity and diabetes. Decreased levels of some lysoPC species in response to AX and RK breads further indicated their beneficial effects on metabolism. Detection of BCAA from the breads to influence insulin secretion warrants a closer consideration regarding protein contents in intervention studies concerning carbohydrate metabolism.
